# Behavioral, Biochemical, and Molecular Indices of Stress are Enhanced in Female Versus Male Rats Experiencing Nicotine Withdrawal

**DOI:** 10.3389/fpsyt.2013.00038

**Published:** 2013-05-20

**Authors:** Oscar V. Torres, Luciana G. Gentil, Luis A. Natividad, Luis M. Carcoba, Laura E. O’Dell

**Affiliations:** ^1^Department of Psychology, The University of Texas at El PasoEl Paso, TX, USA; ^2^Department of Biological Sciences, The University of Texas at El PasoEl Paso, TX, USA

**Keywords:** sex difference, adolescent, adolescence, CRF, nucleus accumbens, tobacco

## Abstract

Stress is a major factor that promotes tobacco use and relapse during withdrawal. Although women are more vulnerable to tobacco use than men, the manner in which stress contributes to tobacco use in women versus men is unclear. Thus, the goal of this study was to compare behavioral and biological indices of stress in male and female rats during nicotine withdrawal. Since the effects of nicotine withdrawal are age-dependent, this study also included adolescent rats. An initial study was conducted to provide comparable nicotine doses across age and sex during nicotine exposure and withdrawal. Rats received sham surgery or an osmotic pump that delivered nicotine. After 14 days of nicotine, the pumps were removed and controls received a sham surgery. Twenty-four hours later, anxiety-like behavior and plasma corticosterone were assessed. The nucleus accumbens (NAcc), amygdala, and hypothalamus were examined for changes in corticotropin-releasing factor (CRF) gene expression. In order to differentiate the effects of nicotine withdrawal from exposure to nicotine, a cohort of rats did not have their pumps removed. The major finding is that during nicotine withdrawal, adult females display higher levels of anxiety-like behavior, plasma corticosterone, and CRF mRNA expression in the NAcc relative to adult males. However, during nicotine exposure, adult males exhibited higher levels of corticosterone and CRF mRNA in the amygdala relative to females. Adolescents displayed less nicotine withdrawal than adults. Moreover, adolescent males displayed an increase in anxiety-like behavior and an up-regulation of CRF mRNA in the amygdala during nicotine exposure and withdrawal. These findings are likely related to stress produced by the high doses of nicotine that were administered to adolescents to produce equivalent levels of cotinine as adults. In conclusion, these findings suggest that intense stress produced by nicotine withdrawal may contribute to tobacco use in women.

## Introduction

Epidemiological reports have indicated that women are more susceptible to tobacco use as compared to men (Perkins, 2009; Lombardi et al., 2011; Rahmanian et al., 2011). For example, women consume more tobacco products relative to men (Hammond, 2009; Oh et al., 2010). Women also exhibit higher relapse rates and are less likely to benefit from nicotine replacement therapy (NRT) than men (Perkins, 2001; Cepeda-Benito et al., 2004; Schnoll et al., 2007; Perkins and Scott, 2008; Piper et al., 2010). During abstinence from tobacco, women also report more intense symptoms of withdrawal than men (Heishman et al., 2010; Nakajima and al’Absi, 2012; Perkins et al., 2013). There is also evidence to suggest that the enhanced susceptibility to tobacco use in women begins at a young age. For example, a recent survey revealed that the daily consumption of tobacco is higher in adolescent females than males [Centers for Disease Control and Prevention (CDC), 2012]. During abstinence from tobacco, adolescent females also report higher levels of stress and relapse rates as compared to adolescent males (Anderson and Burns, 2000; Colby et al., 2000; Dickmann et al., 2009). Regardless of age, females are at a higher risk of developing tobacco-related diseases than males (Langhammer et al., 2000, 2003; Kiyohara and Ohno, 2010). Despite the magnitude of this problem, there is a critical knowledge gap regarding the factors that contribute to enhanced vulnerability to tobacco use among women.

Stress has emerged as a major factor that contributes to tobacco use in women. For example, women report more often than men that the anxiety-reducing effects of cigarettes are the main reason for smoking (Perkins and Scott, 2008; Piper et al., 2010; Perkins et al., 2012). Although tobacco is used to cope with anxiety, long-term tobacco use is also motivated by avoiding negative affective states, such as stress, that emerge during withdrawal (Aronson et al., 2008; Hughes and Callas, 2010; Parrott and Murphy, 2012; Perkins et al., 2012). Accordingly, women also report higher levels of stress during abstinence from tobacco than men (Schnoll et al., 2007; Perkins and Scott, 2008; Xu et al., 2008; Perkins et al., 2012; Saladin et al., 2012). In addition, women display higher levels of cortisol (a biological marker of stress in humans) during tobacco abstinence as compared to men (Hogle and Curtin, 2006). These studies suggest that stress is an important factor that contributes to tobacco use in women.

Pre-clinical evidence has established that the motivational properties of tobacco are due, in large part, to the presence of nicotine. A study comparing sex differences during withdrawal from nicotine demonstrated that female adult rats display more physical signs of nicotine withdrawal relative to males (Hamilton et al., 2009). Also, female adult mice display more anxiety-like behavior on the elevated plus maze during nicotine withdrawal as compared to males (Caldarone et al., 2008). Taken together, there is evidence at the clinical and pre-clinical levels to suggest that females experience higher levels of stress during nicotine withdrawal. However, there are several remaining questions with regard to the underlying neurobiology that modulates the contribution of stress to tobacco use in females.

The main neuroendocrine substrate of the stress response is the hypothalamic-pituitary-adrenal (HPA) axis (see Smith and Vale, 2006; Gallagher et al., 2008). When a stressor is experienced, corticotropin-releasing factor (CRF) is secreted from the hypothalamus that then stimulates adrenocorticotropic hormone (ACTH) release from the pituitary gland. ACTH then simulates the release of corticosterone and other glucocorticoids from the adrenal cortex. Corticosterone serves as a major negative feedback that terminates HPA axis activity. Within the hypothalamus, corticosterone binds to nuclear glucocorticoid receptor II subunits causing an inhibition of CRF mRNA synthesis. Studies comparing biological indices of stress produced by nicotine withdrawal have demonstrated that plasma levels of corticosterone and ACTH are increased in rats experiencing withdrawal from this drug (Rhodes et al., 2004; Semba et al., 2004; Lutfy et al., 2006). With regard to sex differences, female adult rats display elevated plasma levels of corticosterone and ACTH during nicotine withdrawal relative to males (Gentile et al., 2011; Skwara et al., 2012).

Recent theories of drug abuse have suggested that CRF plays a central role in the development of negative affective states that emerge during withdrawal (Koob and Volkow, 2010). Changes in CRF systems are hypothesized to occur within brain structures of the extended amygdala, including the central nucleus of the amygdala, and the nucleus accumbens (NAcc) (Koob, 2010; Bruijnzeel, 2012). Pre-clinical work with nicotine has supported this hypothesis, as CRF-like immunoreactivity is increased in the amygdala during nicotine withdrawal (George et al., 2007). Consistent with this, CRF mRNA levels are over-expressed in the central nucleus of the amygdala during nicotine withdrawal (Aydin et al., 2011). Also, administration of non-specific CRF-R1/R2 receptor antagonists into the amygdala or NAcc have been shown to reverse the deficits in brain reward function produced by nicotine withdrawal (Marcinkiewcz et al., 2009; Bruijnzeel et al., 2012). Collectively, these studies suggest that CRF systems within the NAcc and amygdala play an important role in mediating nicotine withdrawal. To our knowledge; however, no one has examined whether the influence of CRF systems on nicotine withdrawal is sex-dependent.

Thus, the goal of this study was to compare various biological and behavioral indices of stress during nicotine withdrawal in female and male rats. Anxiety-like behavior was examined on the elevated plus maze and open-field tests. Plasma corticosterone levels, and changes in CRF gene expression in the amygdala and NAcc were also explored. CRF gene expression was also examined in the hypothalamus given the primary role of this structure in initiating stress responses. A sub-goal of this study was to examine whether sex differences in adult rats occur during the adolescent period of development. Thus, the biological and behavioral indices of stress produced nicotine withdrawal were also compared in *adolescent* male and female rats. In order to differentiate the effects of withdrawal from those produced by nicotine exposure, a separate cohort of rats from both age and sex groups did not experience withdrawal and were assessed with nicotine circulating in their system. Another important factor to consider when comparing the effects of nicotine across age and sex is differences in metabolism of this drug. Given this potential confound, an initial study was conducted to determine equivalent plasma levels of nicotine in female and male rats of both ages.

## Materials and Methods

### Animals

Male and female adult (*n* = 92) and adolescent (*n* = 98) Wistar rats were used. Rats were bred in the Psychology Department from a stock of out bred Wistar rats from Harlan, Inc. (Indianapolis, IN, USA). All rats were housed in groups of two to three per cage in a humidity- and temperature-controlled (20–22°C) vivarium using a 12-/12-hour light/dark cycle with lights off at 8:00 a.m. The home cages consisted of a rectangular Plexiglas^®^ hanging cage (41.5 cm long × 17 cm wide × 21 cm high) with pine bedding. Rats had *ad libitum* access to standard rodent chow and water at all times except during testing. Adults were postnatal day (PND) 60 and adolescents were PND 28 at the start of the experiment. All rats were handled for approximately 5 min/day for 3 days prior to the start of experimentation. All procedures were approved by the UTEP Animal Care and Use Committee and followed the guidelines of the National Institutes of Health Guide for the Care and Use of Laboratory Animals.

### Nicotine exposure and withdrawal

Rats were anesthetized with an isoflurane/oxygen mixture (1–3% isoflurane) and received a sham surgery or were surgically prepared with subcutaneous pumps that delivered nicotine continuously for 14 days. After 14 days of nicotine exposure, the pumps were surgically removed and control rats received another sham surgery. After pump removal, rats were returned to their home cages. Twenty-four hours later, rats were tested for various behavioral and biological measures of anxiety.

### Study 1: Assessing nicotine metabolism across experimental conditions

Nicotine metabolism was assessed indirectly by comparing cotinine levels in plasma from adolescent and adult male and female rats during exposure and withdrawal from nicotine. Adult rats received pumps that were appropriately sized for larger animals (4.5 mm in length; Alzet model 2ml2), whereas adolescents received either one or two pumps that were approximately half as small (2.5 mm in length; Alzet model 2002). Different doses of nicotine were delivered for 14 days, as described below. Plasma samples were collected from tail blood on days 7, 10, and 14 of nicotine exposure. After 14 days of nicotine exposure, the pumps were surgically removed and plasma samples were collected 6, 12, and 24 h later.

Separate groups of rats were used to determine equivalent doses in adolescent and adult male and female rats. One group of male and female adults was prepared with pumps (model 2ml2) that delivered a nicotine dose of 3.2 mg/kg/day (expressed as base form) that produces robust physical and affective signs of withdrawal in adult rats (O’Dell et al., 2004). Given the fast growth rates and drug metabolism during adolescence, three groups of adolescent rats received pumps with different nicotine doses and experimental procedures. First, a group of male and female adolescents was prepared with one small pump (model 2002) that delivered 4.7 mg/kg/day of nicotine for 14 days. This dose was selected from previous work showing that adolescents implanted with a large pump (model 2ml2) require 1.5-fold higher doses of nicotine to produce equivalent levels in adult rats (O’Dell et al., 2006). Second, a group of male and female adolescents was prepared with one small osmotic pump containing 4.7 mg/kg/day of nicotine. Seven days later, the pump was replaced with a new pump that was re-adjusted for the rats’ rapid weight gain. Last, a third group of male and female adolescents was prepared with two small pumps that each delivered 4.7 mg/kg/day each of nicotine for 14 days. This group received a total of 9.4 mg/kg/day of nicotine. Plasma cotinine levels were analyzed using commercially available 96-well plate ELISA kits (OraSure Technologies, Inc., Bethlehem, PA, USA). Standard curves were used to estimate plasma cotinine levels using a Spectra Maxplus spectrophotometer (Molecular Devices Inc., Sunnyvale, CA, USA).

### Study 2: Assessing behavioral and biological indices of stress during nicotine exposure and withdrawal

Adolescent and adult male and female rats received a sham surgery or were implanted with nicotine pumps (Alzet model 2ml2 for adults and two Alzet models 2002 for adolescents). Adult rats received a nicotine dose of 3.2 mg/kg/day (expressed as base form) for 14 days and adolescent rats received a total nicotine dose of 9.4 mg/kg/day (expressed as base form) for 14 days. To minimize stress produced by repeated tail vein blood sampling from study 1, separate groups of rats were used in this study.

After 14 days of nicotine exposure, the pumps were removed to induce spontaneous withdrawal. Twenty-four hours after pump removal, behavioral tests were conducted to compare physical signs of withdrawal and anxiety-like behavior, using the elevated plus maze and open-field tests. After behavioral testing, the brains were removed and analyzed for CRF mRNA levels using qRT-PCR. Blood samples were also collected and analyzed for corticosterone levels. To examine anxiety-like behavior and biological markers of stress during nicotine exposure, separate cohorts of rats did not have their pumps removed and were tested with nicotine circulating in their system on the 14th day of nicotine exposure. At the time of testing, adult rats were PND 75 and adolescent rats were PND 43.

Rats were tested for anxiety-like behavior using the elevated plus maze procedure. The animals were first acclimated to the testing room in a rectangular Plexiglas^®^ cage for 20 min. After 20 min, the rats were placed onto the elevated plus maze, which was in the middle of the testing room beneath a red light. The plus maze apparatus consisted of four arms (10 cm × 50 cm) that were elevated to a height of 50 cm above the ground. The closed arms had 40 cm high walls around them, and the open arms did not have walls that enclosed the open platforms. At the beginning of the test, the rats were placed into the maze facing the open arm and time spent in each arm was recorded for 5 min. The maze was thoroughly cleaned with 70% ethanol and then water between each individual test. Rats that fell off the maze were excluded from the study.

After elevated plus maze testing, the rats were returned to the isolation cage for 10 min. The open-field apparatus consisted of a clear Plexiglas^®^ box (60 cm × 60 cm × 15 cm) that was positioned in the middle of an adjacent room beneath a red light. The walls of the maze were clear and the floor was divided into 25 equal squares (12 cm × 12 cm; 16 peripheral and 9 center squares). At the start of the test, rats were placed in the center of the open field, and time spent in the center versus corner areas was recorded for 5 min.

After the open-field test, the rats were returned to the isolation cage for somatic signs of withdrawal testing. Ten minutes later, the rats were moved to another testing room and placed in a clear Plexiglas^®^ cylindrical container (30 cm × 29 cm) cage for 10 min. Rats were then monitored for physical signs of nicotine withdrawal for 10 min. The observed signs include blinks, writhes, body shakes, teeth chatters, gasps, and ptosis. If present, ptosis was counted only once. The total number of somatic signs was defined as the sum of individual occurrences of the aforementioned signs during the entire observation period. The duration of the entire test battery was approximately 70 min.

After behavioral testing, rats were sacrificed by rapid decapitation to ensure preservation of the neurochemical environment and minimize degradation during tissue dissection. The amygdala, hypothalamus, and NAcc from both hemispheres were collected and flash frozen at −80°C within an estimated time of 30 s from sacrifice. Total RNA was isolated from neuronal tissue samples using the All Prep DNA/RNA Mini kit (QIAGEN, Inc.) for small tissue sections. After isolation, RNA was quantified using a UV/V spectrophotometer (Beckman Coulter Inc.). The target ratio of 1.8–2.0 for A260/280 was used as an inclusion criterion for all RNA samples. The quality of the RNA was then visualized by MOPS 1% agarose gel (37% formaldehyde) using the Thermo Scientific easy cast electrophoresis system. The gels were verified for characteristic 18S and 28S ribosomal RNA bands using ethidium bromide and the Bio-Rad ChemiDoc XRS+ imaging system. Samples that had insufficient amounts of RNA were excluded from further analyses. One microgram of total RNA was then digested with DNaseI, Amp Grade (Invitrogen) prior to cDNA synthesis in order to remove any DNA contamination. The RNA was then reverse transcribed into cDNA with the Advantage^®^ RT-for-PCR kit (Clontech) using Oligo(dT) primers, following the manufacturer’s instructions. Once the cDNA was synthesized, the cDNA samples were diluted 1:10 in nuclease-free water, separated into aliquots and stored at −20°C. Specific primers for CRF and reference gene ribosomal protein L13A (RPL13A) were obtained from Integrated DNA Technologies, Inc., with amplicons between 71 and 142 base-pairs (see Table 1).

**Table 1:** **Primer sequences**.

Symbol	Forward primer	Reverse primer
CRF	5′ ATGCTGCTGGTGGCTCTGT 3′	5′ GGATCAGAATCGGCTGAGGT 3′
RPL13A	5′ GGATCCCTCCACCCTATGACA 3′	5′ CTGGTACTTCCACCCGACCTC 3′
GAPDH	5′ CAACTCCCTCAAGATTGTCAGCAA 3′	5′ GGCATGGACTGTGGTCATGA 3′
Pol2a	5′ CGTATCCGCATCATGAACAGTGA 3′	5′ TCATCCATCTTATCCACCACCTCTT 3′
Actb	5′ CTATGAGCTGCCTGACGGTC 3′	5′ AGTTTCATGGATGCCACAGG 3′

The rationale for using RPL13A as a reference gene is based upon an initial study examining tissue from a group of adult rats (*n* = 27) that was conducted before quantifying CRF gene expression across experimental groups. Four commonly used reference genes were tested as potential candidates for the normalizing gene, including: actin (Actb), glyceraldehyde-3-phosphate dehydrogenase (GAPDH), RNA polymerase II (Pol2a), and RPL13A. The findings revealed that the expression of RPL13A was the most stable and similar across male and female control and nicotine-treated rats. Based on our results, we believe that expression profiling of normalizing genes is important when employing qRT-PCR techniques involving male and female rats.

Commercially available SYBR^®^ Fast qPCR fluorescent labeling kits (Kapa Biosystems, Inc.) were used to perform qRT-PCR using the Mastercycler ep Realplex2 System (Eppendorf, Inc.). All samples were analyzed in triplicates and amplified by the following protocol: initial denaturing at 95°C for 5 min, continued denaturing at 95°C for 15 s; annealing at 59°C for 15 s; extension at 68°C for 20 s, for a total of 40 cycles. CRF mRNA expression was normalized by RPl-13A mRNA expression using the comparative C_T_ method adopted from Schmittgen and Livak (2008). The amplification specificity for each primer was tested for a single-product, as shown by a single band via TAE 1% gel electrophoresis and visualized on the Bio-Rad ChemiDoc XRS+ system.

Corticosterone levels were assessed in blood samples that were collected from trunk blood during sacrifice. The samples were centrifuged for 15 min at 5,000 × *g* at 4°C. The resultant plasma was then stored at −80°C until analyzed. Corticosterone levels were estimated using a 96-well plate ELISA kit (Assaypro Inc.) using a Spectra Maxplus spectrophotometer (Molecular Devices Inc.).

### Statistical approach

For study 1, cotinine values during nicotine exposure were analyzed using a three-factor mixed model ANOVA with sex (male and female), and age (adult and adolescent) as between subject factors, and day of sampling (7, 10, and 14 days) as a repeated measures factor. Similarly, cotinine values during nicotine withdrawal were analyzed using a three-factor mixed model ANOVA with sex (male and female), and age (adult and adolescent) as between subject factors, and time of sampling (6, 12, and 24 h) as a repeated measures factor. For study 2, each measure was analyzed separately using three-factor ANOVAs with sex (male and female), age (adult and adolescent), and treatment (control, nicotine exposure, and nicotine withdrawal) as between subject factors. In cases where three-way interaction effects were significant, individual group comparisons were reported. However, in cases where three-way interactions were not significant, two-way interactions were reported. All *post hoc* tests were conducted using Fisher’s LSD tests where appropriate (*P* < 0.05). Given that the results revealed interaction effects, main effects were not reported. Thus, interaction effects were reported with *post hoc* tests, and main effects were not included given the interaction effects provide more information about group differences, which was the goal of the paper.

## Results

**Figure 1:**
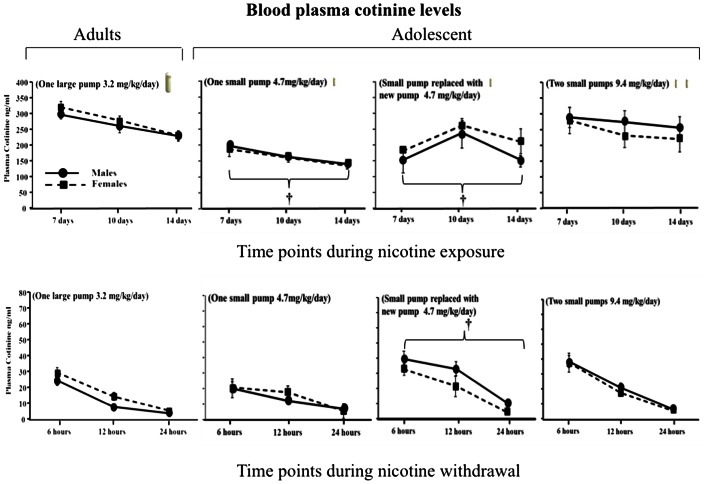
**Blood plasma cotinine levels (ng/ml ± SEM) 7, 10, and 14 days during nicotine exposure (top row) and then 6, 12, and 24 h after pump removal (bottom row) in adult and adolescent male and female rats**. Adult rats (*n* = 26) received a large pump (model 2ml2) that delivered nicotine 3.2 mg/kg for 14 days. Three separate groups of adolescent rats received a smaller model of pump (model 2002) that delivered: (1) a dose of 4.7 mg/kg/day for 14 days (*n* = 17), (2) a dose of 4.7 mg/kg/day that was replaced after 7 days with a new pump that also delivered 4.7 mg/kg/day (*n* = 17), and (3) a dose of 9.4 mg/kg/day that was divided in two small pumps (*n* = 32). The dagger (†) denotes a significant difference across all time points between nicotine-treated adolescents and adults (*P* < 0.05).

Figure 1 illustrates cotinine levels across adolescent and adult male and female rats during nicotine exposure and withdrawal. Regarding sex differences, the results revealed that there were no sex differences in cotinine levels during nicotine exposure [*F*(1, 79) = 0.96, *P* > 0.05] and withdrawal [*F*(1, 84) = 0.19, *P* > 0.05] regardless of the age of the animals. This suggests that sex differences can be appropriately compared across all of the nicotine pump conditions. Regarding age differences during nicotine exposure, adults displayed higher cotinine levels than adolescents prepared with one small pump and adolescents re-implanted with one small pump that was adjusted for weight gain (main effect of treatment) [*F*(3, 79) = 8.96, *P* < 0.05]. However, adult cotinine levels were similar to that of adolescents prepared with two small pumps that each delivered 4.7 mg/kg/day of nicotine for 14 days. A similar pattern was observed during nicotine withdrawal, such that similar levels of cotinine were observed in adults and adolescents that were implanted with two small pumps that each delivered a nicotine dose of 4.7 mg/kg/day. These data suggest that adolescents require two osmotic pumps delivering a total nicotine volume of 9.4 mg/kg/day to produce similar cotinine levels as adults with one pump that delivers 3.2 mg/kg/day.

Table 2 denotes total somatic signs of withdrawal (mean ± SEM) during nicotine exposure and withdrawal in adult and adolescent male and female rats. Somatic signs were analyzed using the total amount of signs elicited during the entire observation period. A three-way analysis of withdrawal signs revealed that there were no interaction effects between sex, age, and treatment [*F*(2, 92) = 0.84, *P* > 0.05]. However, a two-way analysis of withdrawal signs revealed a significant interaction between age and treatment [*F*(2, 92) = 12.08, *P* < 0.05]. Subsequent *post hoc* analyses revealed that adult rats that were tested during nicotine withdrawal displayed an increase in signs of withdrawal compared to their respective controls (*P < 0.05). There were no differences in the magnitude of withdrawal signs across male and female adolescent rats.

**Table 2:** **Physical signs of withdrawal**.

Experimental group	Adult male	Adult female	Adolescent male	Adolescent female
Controls	7.6 ± 0.8	7.8 ± 0.9	5 ± 0.7	3.8 ± 0.3
Nicotine exposure	5.2 ± 0.4	4.3 ± 0.3	3.2 ± 0.3	4.3 ± 0.5
Nicotine withdrawal	*15.5 ± 1.8	*11.5 ± 1.4	3.8 ± 0.3	3.2 ± 0.6

The asterisks (*) denote a significant difference from respective controls (P < 0.05).

**Figure 2:**
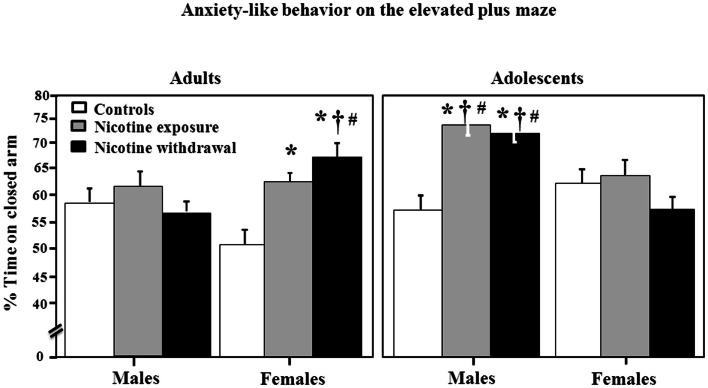
**Percent time spent in the closed arm of the elevated plus maze during nicotine exposure and withdrawal in adult male (control *n* = 13; nicotine exposure *n* = 15; nicotine withdrawal *n* = 9), adult female (control *n* = 10; nicotine exposure *n* = 16; nicotine withdrawal *n* = 13), adolescent male (control *n* = 6; nicotine exposure *n* = 5; nicotine withdrawal *n* = 5), and adolescent female (control *n* = 6; nicotine exposure *n* = 5; nicotine withdrawal *n* = 5) rats**. The asterisks (*) denote a significant difference between nicotine-treated rats and their respective controls, the daggers (†) denote a significant difference between males and females, and the number signs (#) denote a significant difference between adults and adolescents (*P* < 0.05).

Figure 2 illustrates anxiety-like behavior as assessed by the elevated plus maze during nicotine exposure and withdrawal. Anxiety-like behavior was operationally defined as an increase in time spent in the closed arm as compared to controls. A three-way analysis of percent time spent in the closed arm revealed a significant interaction between sex, age, and treatment [*F*(2, 96) = 8.85, *P* < 0.05]. Subsequent *post hoc* analyses revealed that adult females that were tested during nicotine exposure displayed an increase in anxiety-like behavior relative to controls (*P < 0.05). However, adult females tested during nicotine withdrawal displayed an increase in anxiety-like behavior that was significantly higher than their respective controls (*P < 0.05), male counterparts (†*P* < 0.05), and adolescent counterparts (#*P* < 0.05). In adolescents, the males displayed the largest effects of nicotine exposure and withdrawal on anxiety-like behavior as compared to respective controls (*P < 0.05), female counterparts (†*P* < 0.05), and adolescent counterparts (#*P* < 0.05).

**Figure 3:**
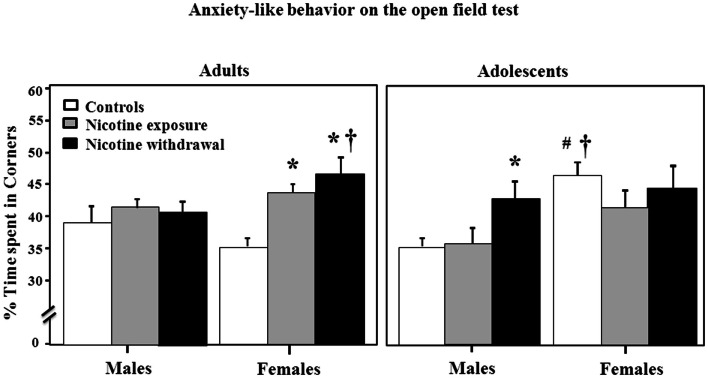
**Percent time spent in the corner areas in the open-field testing during nicotine exposure and withdrawal in adult male (control *n* = 9; nicotine exposure *n* = 15; nicotine withdrawal *n* = 10), adult female (control *n* = 10; nicotine exposure *n* = 16; nicotine withdrawal *n* = 13), adolescent male (control *n* = 6; nicotine exposure *n* = 5; nicotine withdrawal *n* = 5), and adolescent female (control *n* = 5; nicotine exposure *n* = 5; nicotine withdrawal *n* = 5) rats**. The asterisks (*) denote a significant difference between nicotine-treated rats and their respective controls, the daggers (†) denote a significant difference between males and females, and the number sign (#) denotes a significant difference between adults and adolescents (*P* < 0.05).

Figure 3 illustrates anxiety-like behavior as assessed by the open-field test during nicotine exposure and withdrawal. Anxiety-like behavior was operationally defined as an increase in time spent in the corners of the open field as compared to controls. A three-way analysis of percent corner time revealed a significant interaction between sex, age, and treatment [*F*(2, 92) = 3.85, *P* < 0.05]. Subsequent *post hoc* analyses revealed that adult females tested during nicotine exposure displayed an increase in anxiety-like behavior relative to controls (*P < 0.05). However, adult females tested during nicotine withdrawal displayed an increase in anxiety-like behavior that was higher than respective controls (*P < 0.05) and their male counterparts (†*P* < 0.05). In adolescents, males tested during nicotine withdrawal displayed an increase in anxiety-like behavior relative to controls (*P < 0.05). Adolescent female controls displayed an increase in anxiety-like behavior relative to males (†*P* < 0.05) and their adult counterparts (#*P* < 0.05).

**Figure 4:**
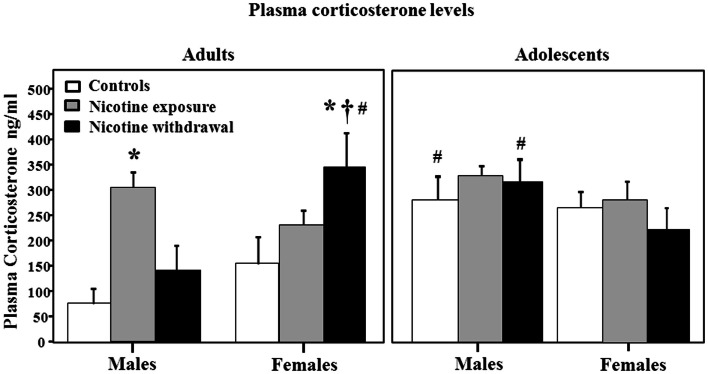
**Plasma corticosterone levels during nicotine exposure and withdrawal in adult male (control *n* = 7; nicotine exposure *n* = 9; nicotine withdrawal *n* = 6), adult female (control *n* = 8; nicotine exposure *n* = 8; nicotine withdrawal *n* = 8), adolescent male (control *n* = 6; nicotine exposure *n* = 5; nicotine withdrawal *n* = 5), and adolescent female (control *n* = 6; nicotine exposure *n* = 5; nicotine withdrawal *n* = 5) rats**. The asterisks (*) denote a significant difference between nicotine-treated male and female adult rats and their respective controls, the dagger (†) denotes a significant difference between males and females, and the number signs (#) denote a significant difference between adolescents and adults (*P* < 0.05).

Figure 4 illustrates plasma corticosterone levels during nicotine exposure and withdrawal. A three-way analysis of corticosterone levels revealed a significant interaction between sex, age, and treatment [*F*(2, 66) = 3.2, *P* < 0.05]. Subsequent *post hoc* analyses revealed that adult males tested during nicotine exposure displayed an increase in corticosterone levels relative to controls (*P < 0.05). Adult females tested during nicotine withdrawal displayed an increase in corticosterone levels relative to controls (*P < 0.05), male counterparts (†*P* < 0.05), and adolescent counterparts (#*P* < 0.05). In adolescents, the male controls and males tested during nicotine withdrawal displayed an increase in corticosterone levels relative to their adult counterparts (#*P* < 0.05).

**Figure 5:**
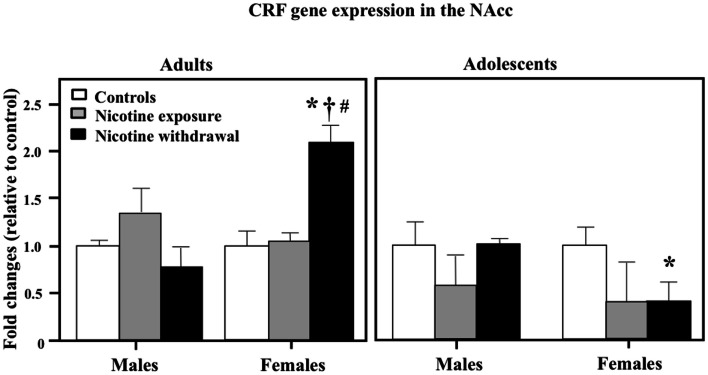
**CRF gene expression in the NAcc during nicotine exposure and withdrawal in adult male (control *n* = 4; nicotine exposure *n* = 4; nicotine withdrawal *n* = 4), adult female (control *n* = 4; nicotine exposure *n* = 4; nicotine withdrawal *n* = 4), adolescent male (control *n* = 6; nicotine exposure *n* = 5; nicotine withdrawal *n* = 5), and adolescent female (control *n* = 5; nicotine exposure *n* = 4; nicotine withdrawal *n* = 5) rats**. The asterisks (*) denote a significant difference between nicotine-treated rats and their respective female controls, the dagger (†) denotes a significant difference between male and female rats, and the number sign (#) denotes a significant difference between adolescent and adult rats (*P* < 0.05).

Figure 5 illustrates CRF gene expression in the NAcc during nicotine exposure and withdrawal. A three-way analysis of CRF gene expression revealed a significant interaction between sex, age, and treatment in this brain region [*F*(2, 42) = 4.34, *P* < 0.05]. Subsequent *post hoc* analyses revealed that adult females tested during nicotine withdrawal displayed an increase in CRF gene expression relative to controls (*P < 0.05), male counterparts (†*P* < 0.05), and adolescent counterparts (#*P* < 0.05). In adolescents, females tested during nicotine withdrawal displayed a decrease in CRF gene expression relative to controls (*P < 0.05).

**Figure 6:**
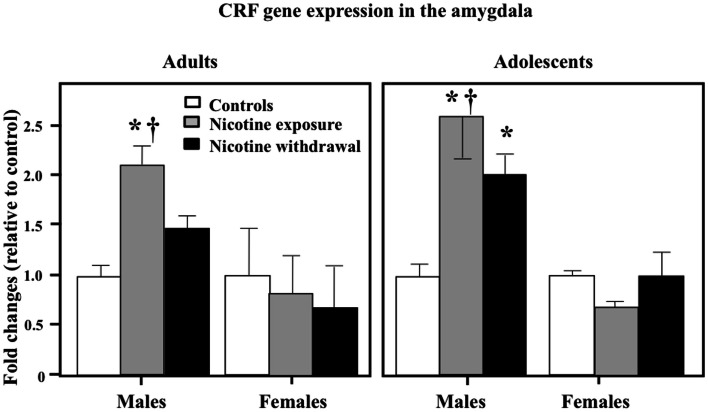
**CRF gene expression in the amygdala during nicotine exposure and withdrawal in adult male (control *n* = 10; nicotine exposure *n* = 5; nicotine withdrawal *n* = 5), adult female (control *n* = 7; nicotine exposure *n* = 7; nicotine withdrawal *n* = 4), adolescent male (control *n* = 4; nicotine exposure *n* = 6; nicotine withdrawal *n* = 4), and adolescent female (control *n* = 4; nicotine exposure *n* = 4; nicotine withdrawal *n* = 4) rats**. The asterisks (*) denote a significant difference between nicotine-treated rats and their respective male controls (*P* < 0.05).

Figure 6 illustrates CRF gene expression in the amygdala during nicotine exposure and withdrawal. A three-way analysis of CRF gene expression revealed that there were no interaction effects between sex, age, and treatment in this brain region [*F*(2, 52) = 0.21, *P* > 0.05]. However, a two-way analysis of CRF gene expression in the amygdala revealed a significant interaction between sex and treatment [*F*(2, 52) = 3.72, *P* < 0.05]. Subsequent *post hoc* analyses revealed that adult and adolescent male rats tested during nicotine exposure displayed a significant increase in CRF gene expression as compared to controls (*P < 0.05) and female counterparts (†*P* < 0.05). In addition, adolescent males tested during nicotine withdrawal displayed an increase in CRF gene expression relative to controls (*P < 0.05).

**Figure 7:**
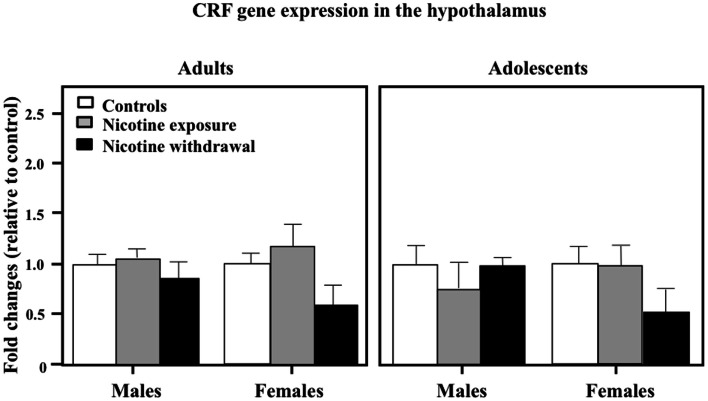
**CRF gene expression in the hypothalamus during nicotine exposure and withdrawal in adult male (control *n* = 13; nicotine exposure *n* = 13; nicotine withdrawal *n* = 6), adult female (control *n* = 6; nicotine exposure *n* = 6; nicotine withdrawal *n* = 4), adolescent male (control *n* = 8; nicotine exposure *n* = 5; nicotine withdrawal *n* = 5), and adolescent female (control *n* = 5; nicotine exposure *n* = 5; nicotine withdrawal *n* = 6) rats**.

Figure 7 illustrates CRF gene expression in the hypothalamus during nicotine exposure and withdrawal. A three-way analysis of CRF gene expression revealed that there were no interaction effects between sex, age, and treatment in this brain region [*F*(2, 68) = 0.02, *P* > 0.05]. Also, a two-way analysis of CRF gene expression revealed that there were no interaction effects between sex and treatment [*F*(2, 68) = 1.27, *P* > 0.05] or age and treatment [*F*(2, 68) = 0.41, *P* > 0.05].

## Discussion

To summarize, during nicotine withdrawal, adult females displayed increases in anxiety-like behavior, increases in plasma corticosterone levels, and changes in CRF gene expression in the NAcc that were higher as compared to males. Control studies comparing sex differences during nicotine exposure, revealed that adult males displayed an increase in plasma corticosterone levels and increases in CRF gene expression in the amygdala. The sex differences in adults did not appear to be confounded by nicotine metabolism, since cotinine values were the same in male and female rats throughout our experimental procedures. Regarding age differences, adolescent males displayed some indices of stress during nicotine exposure that persisted into the withdrawal period. This may have been related to the high doses of nicotine that the adolescents required to produce comparable cotinine values as adults.

The major finding of this study is that adult females experience greater behavioral and biological indices of stress during nicotine withdrawal as compared to males. Adult females spent more time on the closed arm of the elevated plus maze during nicotine withdrawal as compared to males. Consistent with this, adult females also spent more time in the corner areas of the open field during nicotine withdrawal relative to males. Our behavioral results corroborate with our biological assessment of stress, as adult females also displayed increases in plasma corticosterone levels during nicotine withdrawal that were higher than males. Our results are consistent with previous reports demonstrating that female adult mice display more anxiety-like behavior on the elevated plus maze during nicotine withdrawal as compared to males (Kota et al., 2007, 2008; Caldarone et al., 2008). Two recent reports also showed that adult female rats display higher plasma corticosterone levels during nicotine withdrawal as compared to males (Gentile et al., 2011; Skwara et al., 2012).

The present study also revealed that adult females displayed an increase in CRF mRNA expression in the NAcc during nicotine withdrawal that was higher than males. Previous reports support the role of the NAcc in modulating stress. For example, intra-NAcc administration of CRF has been shown to produce anxiety-like behavior on the elevated plus maze (Chen et al., 2012). The NAcc is also strongly activated following presentation of a stressful stimulus (Noh et al., 2012). The latter report showed that the NAcc was activated to a greater extent following restraint stress as compared to cold-water submersion. Thus, the NAcc may be differentially responsive to various types of stressors. Our findings suggest that the NAcc is also involved in stress produced by nicotine withdrawal. Consistent with this hypothesis, the deficits in brain reward function produced by nicotine withdrawal are alleviated by blockade of CRF receptors in the NAcc (Marcinkiewcz et al., 2009). Our finding that the hypothalamus was not altered during withdrawal, is consistent with the finding that CRF mRNA was not altered in the hypothalamus of male rats experiencing spontaneous nicotine withdrawal (Semba et al., 2004). Thus, the hypothalamus may not play a central role in modulating negative affective states involving stress produced by nicotine withdrawal.

Our findings also suggest that the NAcc is a structure involved in sex-dependent differences to drug withdrawal. This is consistent with previous studies examining withdrawal from other drugs of abuse. For example, morphine withdrawal produced a decrease in μ-opioid receptors in the NAcc of female but not male mice (Diaz et al., 2006). Also, multiple withdrawal periods from ethanol produced an increase in proteins involved in vesicular packaging and exocytosis in the NAcc of female but not male rats (Bell et al., 2006, 2009). Following abstinence from cocaine self-administration, delta opioid receptors and dopamine phosphoproteins are increased to a greater extent in the NAcc of female versus male rats (Lynch et al., 2007; Ambrose-Lanci et al., 2008). Taken together with the present findings, there is strong evidence to suggest that the NAcc modulates sex differences produced by withdrawal from drugs of abuse.

There are several ways in which females may be more susceptible to stress produced by nicotine withdrawal. There is much evidence to suggest that CRF systems are enhanced in females versus males (Bangasser, 2013; Valentino et al., 2013). Females display hypersecretion of CRF and more CRF-1 receptors in the locus coeruleus, a brain region that coordinates arousal components of the stress response (Curtis et al., 2006; Bangasser et al., 2013). Females also display a higher ratio of CRF-1 receptors to coupling of G-proteins versus male rats, suggesting that the female CRF system has greater intracellular signaling capacity (Bangasser et al., 2010). The beta-arrestin2 protein is an intracellular protein that internalizes the CRF-1 receptor into the cell cytoplasm and prevents it from being activated by CRF (Aguilera et al., 2004; Holmes et al., 2006). Female rats display lower levels of beta-arrestin2 than male rats, suggesting that females are more responsive to CRF stimulation due to reduced internalization of the CRF-1 receptor as compared to males (Bangasser and Valentino, 2012). Females may also be more susceptible to stress produced by withdrawal via ovarian hormones. For example, direct activation of estrogen-beta receptors (ERβ) increase CRF mRNA expression *in vitro* (Chen et al., 2008; Lalmansingh and Uht, 2008; Zhu and Zhou, 2008). Furthermore, the estrogen gene sequence serves as a promoter of CRF gene transcription (Vamvakopoulos and Chrousos, 1993). Collectively, these studies suggest that females have a hypersensitive CRF system, and this may contribute to the enhanced stress produced by nicotine withdrawal in females versus males.

The present study also revealed a robust increase in CRF gene expression in the amygdala of male rats during nicotine exposure. A recent report showed that CRF levels were increased in the amygdala of adult male rats experiencing nicotine withdrawal (George et al., 2007). The rats in the latter study received a nicotine antagonist to precipitate withdrawal while nicotine was being delivered via an osmotic pump. The findings from the present study are consistent with those of George et al. (2007), given that the rats from both studies had circulating levels of nicotine in their system at the time of analysis. Thus, the possibility exists that nicotine directly activates CRF systems in the amygdala, especially given that the changes in CRF were not observed in the absence of nicotine in our study. Future studies are needed to fully understand the role of CRF systems in the amygdala in modulating the direct effects of nicotine and the long-term consequences of withdrawal from this drug. A unique challenge for this work is that nicotine exposure is an inherent part of studies that assess withdrawal, either by spontaneous or precipitated methods.

The present study also compared sex differences in the somatic signs of nicotine withdrawal. Our findings suggest that there were no differences in somatic signs of withdrawal between adult male and female rats. A report by Hamilton et al. (2009) showed that female rats display more somatic signs of withdrawal relative to males. The discrepancy between these reports may be related to differences in lighting conditions given that Hamilton et al. only reported sex differences in rats that were tested in dim, but not well-lit conditions. In the present study, the somatic signs data were collected in well-lit conditions whereas the anxiety-like behavior was collected in the dark under a red light. Perhaps different lighting conditions may be considered in future studies examining anxiety-like behavior produced by nicotine withdrawal, especially given the reported effects of lighting conditions on the somatic signs of withdrawal.

Another finding of this study is that male and female adolescents generally displayed fewer somatic signs of withdrawal as compared to adults. These findings are consistent with previous work in our laboratory and others demonstrating that the behavioral and neurochemical effects of withdrawal are diminished in adolescent versus adult rats (Smith et al., 2006; Wilmouth and Spear, 2006; Shram et al., 2008; O’Dell, 2009). This study extends this work by showing that adolescent females are also less sensitive to nicotine withdrawal as compared to adult females. An important caveat; however, is that adolescent males displayed anxiety-like behavior and biological markers of stress during nicotine exposure that persisted 24-h later into withdrawal. There are two possible explanations for this effect. First, adolescent males may not be impervious to all aspects of withdrawal, which may induce a stress response that contributes to tobacco use in adolescent males. Second, it is possible that nicotine elicited a stress response in adolescent males. This explanation is consistent with the finding that CRF gene expression was increased in the amygdala of adolescent males during nicotine exposure. We suggest that the ability of nicotine to induce a stress response was likely related to the three-fold higher doses of this drug that were used to produce equivalent plasma levels of cotinine as adults. The lack of stress effects in female adolescents was likely related to high tolerance to the aversive effects of nicotine, an effect that has been previously demonstrated (Torres et al., 2009). Future studies are needed to examine sex differences to stress produced by nicotine withdrawal, perhaps with a model such as nicotine vapor inhalation that circumvents the dosing issues that arose in the present study with osmotic pumps. Despite this, the present study provided important parametric information regarding equivalent doses of nicotine in adolescent and adult rats using different pump sizes. Our results raise an important issue for future studies comparing developmental differences to nicotine since high doses of nicotine may produce stress in adolescent males.

There are some limitations in the present study. In some cases, our behavioral and biochemical measures appear to contradict each other. For example, in adolescent males, we observed an increase in anxiety-like behavior in the plus maze but not the open field. This discrepancy is likely related to the sensitivity of these measures in assessing anxiety-like behavior. In adult females, during withdrawal, the pattern of changes was consistent (high anxiety-like behavior and corticosterone). However, during nicotine exposure the pattern of changes was not consistent (high anxiety-like behavior but no changes in corticosterone). The lack of changes in corticosterone was likely due to a higher baseline value in adult females. In adult males, during withdrawal, the pattern of changes was consistent (no anxiety-like behavior and no changes in corticosterone). However, during nicotine exposure, the pattern of changes was not consistent (no changes in anxiety-like behavior and an increase in corticosterone). One might argue that the changes in corticosterone were aberrant; however, this group also showed an increase in CRF gene expression in the amygdala. Thus, it may be the case the nicotine exposure is more stress inducing in adult males as compared to withdrawal from this drug.

In conclusion, our results suggest that during nicotine withdrawal female rats display behavioral and biological markers of stress that are enhanced compared to males. These findings contribute to a body of literature showing that female rats display greater rewarding effects of nicotine as compared to males (Donny et al., 2000; Klein et al., 2004; Chaudhri et al., 2005; Torres et al., 2009). Taken together, there is pre-clinical evidence to suggest that enhanced rewarding effects of nicotine and intense stress produced by withdrawal both contribute to the greater vulnerability to tobacco use observed in women. In addition, our findings suggest that the most effective cessation treatments for women should also alleviate intense stress produced by nicotine withdrawal. For example, one approach might include CRF antagonists in combination with other tobacco cessation treatments, such as NRT or partial nicotinic agonists. Future studies are needed to understand the complex interactions in the brain that modulate sex differences to nicotine use. This work is important toward reducing health disparities related to tobacco use among women.

## Conflict of Interest Statement

The authors declare that the research was conducted in the absence of any commercial or financial relationships that could be construed as a potential conflict of interest.
